# Static Disorder in Lead Halide Perovskites

**DOI:** 10.1021/acs.jpclett.2c01652

**Published:** 2022-08-02

**Authors:** Stefan Zeiske, Oskar J. Sandberg, Nasim Zarrabi, Christian M. Wolff, Meysam Raoufi, Francisco Peña-Camargo, Emilio Gutierrez-Partida, Paul Meredith, Martin Stolterfoht, Ardalan Armin

**Affiliations:** † Sustainable Advanced Materials (Sêr-SAM), Department of Physics, Swansea University, Singleton Park, Swansea SA2 8PP, United Kingdom; ‡ EPFL STI IEM PV-LAB, Rue de la Maladière 71b, CH-2002 Neuchâtel 2, Switzerland; § Soft Matter Physics Institute of Physics and Astronomy, University Potsdam, Karl-Liebknecht-Straße 24-25, 14476 Potsdam-Golm, Germany

## Abstract

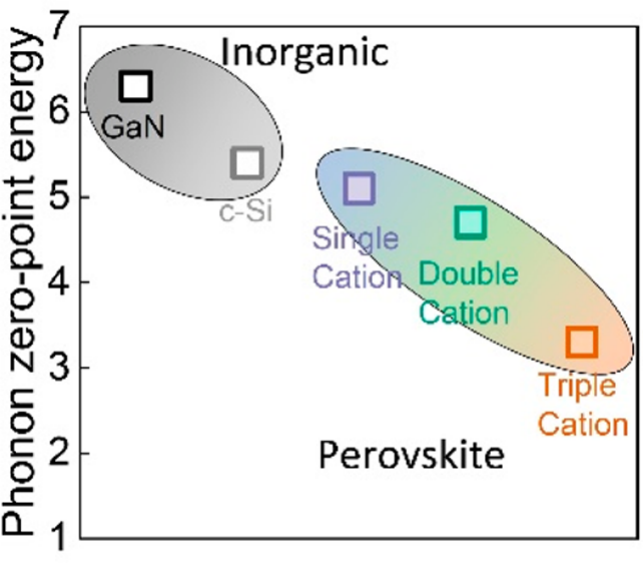

In crystalline and amorphous semiconductors, the temperature-dependent Urbach energy can be determined from the inverse slope of the logarithm of the absorption spectrum and reflects the static and dynamic energetic disorder. Using recent advances in the sensitivity of photocurrent spectroscopy methods, we elucidate the temperature-dependent Urbach energy in lead halide perovskites containing different numbers of cation components. We find Urbach energies at room temperature to be 13.0 ± 1.0, 13.2 ± 1.0, and 13.5 ± 1.0 meV for single, double, and triple cation perovskite. Static, temperature-independent contributions to the Urbach energy are found to be as low as 5.1 ± 0.5, 4.7 ± 0.3, and 3.3 ± 0.9 meV for the same systems. Our results suggest that, at a low temperature, the dominant static disorder in perovskites is derived from zero-point phonon energy rather than structural disorder. This is unusual for solution-processed semiconductors but broadens the potential application of perovskites further to quantum electronics and devices.

Light absorption by semiconductors near and below their energy gap provides information about the density of states and subgap states, such as traps,^1,2^ tail states, and intermolecular species.^3,4^ In banded semiconductors, the subgap features in the absorption spectrum can be divided into two regions: (i) the band-edge region with a finite width determined by tail states induced by energetic disorder^5^ and (ii) the subgap region as a result of the photoexcitations via deep trap states with distinct absorption features several orders of magnitude weaker than the absorption onset.^6,7^ The disorder-induced tail state absorption in the band-edge region usually shows an exponential dependency with decreasing photon energy (*E*), with the corresponding absorption coefficient (α) being of the form
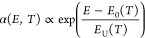
1where *T* is the temperature, *E*_0_ is an onset energy related to the bandgap, and *E*_U_ is the so-called Urbach energy describing the steepness of the exponential decay. The Urbach energy is typically used as a proxy for the degree of energetic disorder, which generally increases with the temperature as a result of the contribution from carrier-phonon interactions. Electronic properties, such as charge carrier mobility and lifetime in semiconductors, are related to the exponential tail states and, thereby, to the Urbach energy. As such, measuring and understanding subgap absorption features in general and, in particular, Urbach energies provides critical information about the electrical and optical properties of the materials.

Lead halide perovskites have emerged as high-efficiency photovoltaic materials in the past decade and are now frequently used in next-generation optoelectronic devices, including light-emitting diodes,^8,9^ solar cells,^10,11^ and photodetectors.^12,13^ These materials are low-temperature vapor-deposited or solution-processed direct bandgap semiconductors that exhibit very sharp absorption onsets,^14^ which are rather atypical for such materials. It has been reported that the static disorder (the low-temperature component of the Urbach energy) in perovskites can be even smaller than for crystalline silicon or epitaxially grown III–V compound semiconductors, such as GaAs.^15^ On the other hand, from sensitive external quantum efficiency (EQE) measurements, the contribution of deep trap states in the subgap absorption has also been observed in these materials and assigned to different trap levels. In two recent contributions, deep trap states were shown to be predominantly present at the perovskite/C_60_ interface (with spectral features manipulated by cavity interference), causing extremely small but detectable photocurrents.^2^ Furthermore, it has been observed that even neat perovskite films contain a certain degree of gap states, indicating the presence of bulk defects.^6,39^ However, the origin and role of these defect states^16−18^ in perovskite semiconductors is still the subject of some debate.^19,20^

Benefiting from recent advancements in the sensitivity of photocurrent spectroscopy,^7^ in this work, we revisit subgap absorption measurements with improved accuracy on state-of-the-art perovskite solar cells based on lead halides with different numbers of cation components. First, we demonstrate that the spectral line shape of absorption features associated with deep trap states are strongly influenced by thickness-dependent optical cavity effects. Hence, the corresponding spectra, if uncorrected, provide no information about the energetic distribution of subgap trap states. Second, we show that the determination of the Urbach energy is limited to an uncertainty of 1 meV as a result of inevitable variations in subgap absorption features caused by said cavity effects. Finally, in light of this, we determine the Urbach energy using temperature-dependent, ultrasensitive EQE measurements. The corresponding static, temperature-independent contributions of the Urbach energy are found to be 3.3 ± 0.9, 4.7 ± 0.3, and 5.1 ± 0.5 meV for triple, double, and single cation perovskite solar cells, respectively. Defect states with small trap signatures observed in sensitive subgap EQE are found not to dominate the perovskite static disorder. Instead, the static disorder in perovskites is only limited by the quantum noise motion of the phonons (zero-point phonon energy), which broadens their potential applicability for realizing quantum devices operating at cryogenic temperatures.

Figure 1a shows the current–voltage (*J*–*V*) characteristics of single, double, and triple cation perovskite solar cells (p-i-n structure) under artificial 1 sun illumination (AM 1.5G condition) exhibiting power conversion efficiencies (PCEs) of 19.1, 21.2, and 19.8%, respectively. The single cation system is composed of methylammonium lead iodide (MAPbI_3_); the double cation is FA_0.93_MA_0.07_PbI_3_; and the triple cation is Cs_0.05_(FA_0.83_MA_0.17_)_0.95_Pb(I_0.83_Br_0.17_)_3_. Detailed information concerning the device fabrication is provided in the Supporting Information. The inset in Figure 1a shows the corresponding EQE spectra (left axis, solid lines) revealing different bandgaps among the three perovskite devices, and EQE-integrated photocurrent densities (right axis, dashed lines) deviate less than 5% from the measured short-circuit current densities, confirming the validity of the *J*–*V* analysis.

**Figure 1:**
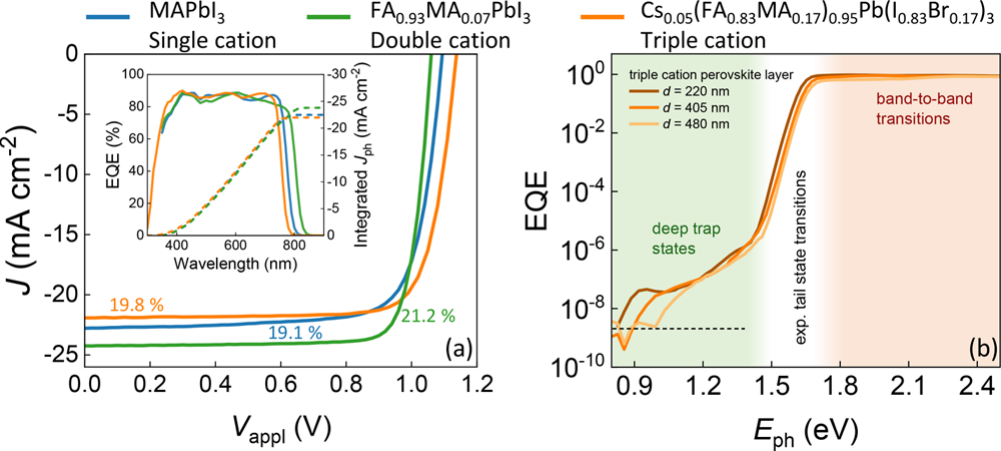
(a) Current–density characteristics of single (MAPbI_3_), double (FA_0.93_MA_0.07_PbI_3_), and triple [Cs_0.05_(FA_0.83_MA_0.17_)_0.95_Pb(I_0.83_Br_0.17_)_3_] cation perovskite solar cells under 1 sun AM 1.5G conditions with power conversion efficiencies indicated in the plot. The inset shows the corresponding EQE spectra (left axis, solid lines) of the three systems along with the EQE-integrated photocurrent *J*_ph_ (right axis, dashed lines). (b) EQE spectra of triple cation perovskite solar cells having different perovskite layer thicknesses. The vertical, dashed line indicates the EQE noise floor, while the inset shows the corresponding apparent Urbach energy spectra in the exponential region as determined by eq 2. A thickness dependence is seen leading to an uncertainty in Urbach energy (*E*_U_) of ±1 meV (see the inset, gray shadowed area).

To measure the subgap EQE regime of the perovskite devices accurately, a recently introduced approach^7^ with enhanced EQE sensitivity of up to −100 dB (corresponding to an EQE of 10^–10^) was used. The wide dynamic range of ultrasensitive EQE presents a clear advantage over optical absorption measurements (which are typically limited to a dynamic range of 20–40 dB), allowing for the Urbach energy to be determined with an unprecedented accuracy. Furthermore, as EQE probes the generation of free charge carriers, effects of strongly bound excitons, if present, are expected to be negligible. Figure 1b shows the EQE spectrum of a triple cation perovskite solar cell plotted as a function of photon energy and compared for different perovskite active layer thicknesses. From these wide dynamic-range EQE spectra, three distinct regions can be identified: (i) the above-gap region, where band-to-band transitions occur and EQE signals are near unity (red-shaded area in Figure 1b), (ii) the exponential tail region, where the EQE decreases exponentially with decreasing energy down to EQE signals as small as 10^–5^ (white-shaded area), and (iii) a region with low-energy subgap features below EQEs of 10^–6^ (green-shaded area).

From the exponential tail region, the Urbach energy can be determined.^21^ For weak absorption (i.e., at energies below the bandgap with α*d* ≪ 1, where *d* is the active layer thickness), we expect α*d* to be directly proportional to the EQE if interference effects are assumed negligible. Under these conditions, the apparent Urbach energy can be subsequently determined from the subgap EQE spectrum via^22^
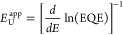
2The (energy-dependent) apparent Urbach energy spectra *E*_U_^app^(*E*) for the triple cation perovskite solar cells, as obtained using eq 2, are shown in the inset in Figure 1b. The Urbach energy (*E*_U_) is determined from the exponential EQE region, where the apparent Urbach energy flattens out. We note, however, that the range of the exponential region, where a flat *E*_U_^app^ is expected, is limited to approximately 0.1 eV, and the estimated Urbach energies in this region vary by approximately 1 meV depending upon the active layer thickness (gray band in Figure 1b). The corresponding *E*_U_ for the triple cation perovskite device was found to be 13.5 ± 1 meV.

At photon energies below the exponential region, additional subgap features, located well above the noise floor (vertical, dashed line in Figure 1b), prevail. Such subgap features have been observed previously in perovskites^10,23^ and recently also in organic semiconductors,^24,25^ being universally assigned to deep trap states. The mechanism of light absorption and charge generation via such trap and impurity states can be understood in terms of optical release of trapped charge carriers, which is the inverse process of Shockley–Read–Hall recombination and generally expected to be dominated by deep (close to midgap) states. It is, however, evident from Figure 1b that the spectral subgap line shape induced by deep trap states varies strongly with active layer thickness (see also Figure S1 of the Supporting Information), pointing toward a strong presence of low-finesse cavity interference effects modulating the EQE spectra in this region.^4,26^

To verify that the thickness dependence of the subgap EQE in the exponential tail and deep trap state region is caused by cavity effects, the role of optical interference in the EQE and apparent Urbach energy spectrum are investigated through optical simulations based on a transfer matrix model.^27^ For this purpose, we assume realistic values for the refractive index *n* (on the basis of spectroscopic ellipsometry) and the extinction coefficient *k* in the active layer. To simulate the subgap absorption, we assume *k* below the bandgap to be composed of a well-defined Urbach energy of 13 meV (representing tail states) and a much broader exponential tail (representing deep trap states) (see Figure S2a of the Supporting Information). The optical constants are then used to simulate the corresponding EQE spectra of solar cells with a device architecture equivalent to those devices used for experiments.

Figure 2 shows the simulated EQE (left axis, solid lines) and reflection spectra (right axis, dashed lines) from the modeled solar cell stack for different active layer thicknesses. In a similar manner to the experimental results shown in Figure 1b, the modeled subgap EQE spectral line shapes in the deep trap state region vary with active layer thickness, with spectral features correlating with the reflection spectra (see also Figure S2b of the Supporting Information), thus confirming that optical interference does affect the subgap EQE. The inset in Figure 2 shows the corresponding apparent Urbach energy spectra confirming an uncertainty of 1 meV in Urbach energy as a result of optical cavity effects and, thus, being in excellent agreement with uncertainty values obtained from the experimental data (see the inset in Figure 1b).

**Figure 2:**
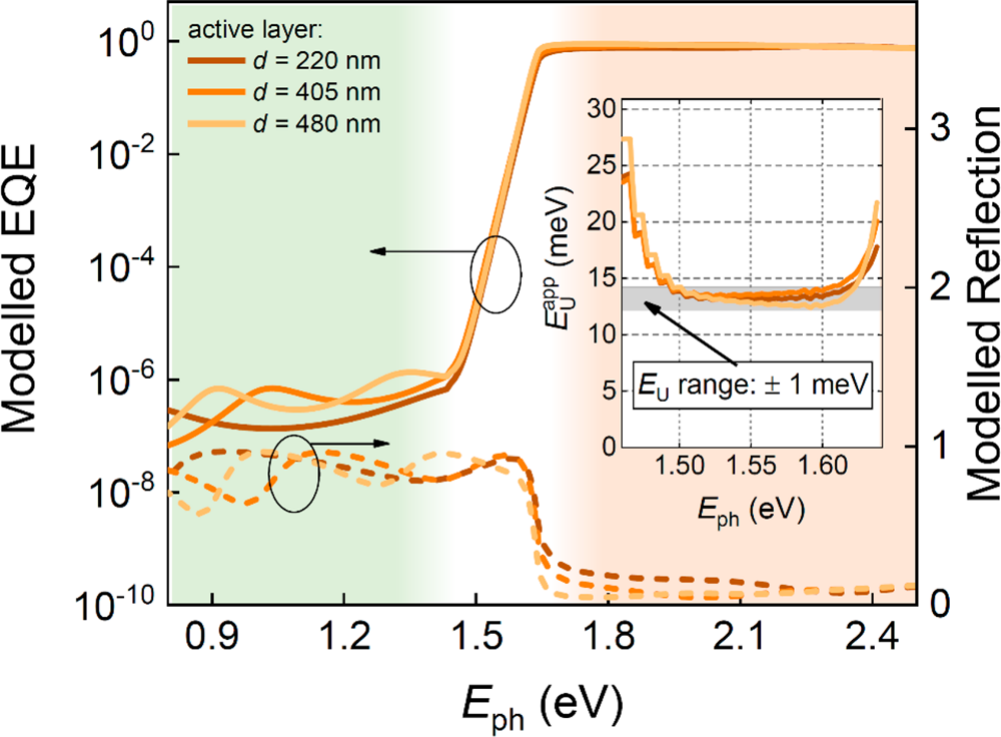
Modeled EQE (left axis, solid lines) and reflection spectra (right axis, dashed lines) plotted as a function of photon energy and compared for different active layer thicknesses. The inset shows the corresponding apparent Urbach energy spectra *E*_U_^app^(*E*) in the exponential region, with cavity effects resulting in a weak thickness-dependent Urbach energy variation of ±1 meV.

From this analysis, two important conclusions can be drawn. First, the spectral line shape of the deep trap region is heavily influenced by optical inference, and thus, one cannot obtain useful information about the intrinsic spectral shape of these states directly from the observed peaks without additional correction. However, this would require knowledge of the spatial distribution of the deep traps in the perovskite film. Because the spatial trap distribution is unknown, the recently introduced methodology of Kaiser et al.^26^ (which is based on inverse transfer matrix analyses and could, in principle, correct for the influence of optical interference) is not applicable here. Second, the Urbach energy can be determined within an uncertainty of 1 meV from the solar cell EQEs if the noise floor is below the tail state spectral region (see Figure 1b, where the sensitivity of the EQE is −90 dB, hence 40 dB below the exponential tail region).

By appreciating the uncertainty limit of the determination of *E*_U_, the temperature dependence of the Urbach energy can be clarified by conducting temperature-dependent, ultrasensitive EQE measurements. We note that the temperature regimes were chosen in such a way that no perovskite lattice phase transitions were present^28−30^ and the apparent Urbach energy spectra in the bandgap regimes were not limited by the monochromaticity imperfection of our EQE apparatus (see Figure S3 of the Supporting Information). Experimentally obtained EQE spectra of all perovskite solar cells along with their corresponding apparent Urbach energy spectra are provided in Figure S4 of the Supporting Information. Urbach energies at room temperature (RT) are found to be 13.0 ± 1.0, 13.2 ± 1.0, and 13.5 ± 1.0 meV for single, double, and triple cation systems, respectively. These values are close to values reported for perovskite-based solar cells in previous studies.^15,23,30−33^

Panels a–c of Figure 3 show the experimentally obtained Urbach energies (symbols) for the three perovskite systems plotted as a function of the temperature. The temperature-dependent behavior *E*_U_(*T*) is found to be described well by the following model:^34,35^
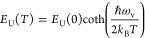
3where ω_v_ is a characteristic phonon frequency, ℏ is the reduced Planck constant, and *k*_B_ is the Boltzmann constant. With σ_0_ = ℏω_v_/2*E*_U_(0) being a temperature-independent constant expected to be of order of unity, eq 3 can be rewritten as *E*_U_(*T*) = *E*_U_(0)coth(σ_0_*E*_U_(0)/*k*_B_*T*). Hence, we expect *E*_U_(*T*) → *k*_B_*T*/σ_0_ at high temperatures. At low temperatures, on the other hand, *E*_U_(*T*) ≈ *E*_U_(0), becoming independent of *T*, where *E*_U_(0) can be understood as a temperature-independent contribution to the Urbach energy reflecting the level of static disorder. The fittings of the experimental *E*_U_(*T*) to eq 3 are shown by solid lines in panels a–c of Figure 3; the corresponding fit parameters are summarized in Table 1. Static contributions to the Urbach energy in the low-temperature limit were found to be as low as 5.1 ± 0.5 meV (single cation), 4.7 ± 0.3 meV (double cation), and 3.3 ± 0.9 meV (triple cation). As such, these values are comparable to those of other crystalline semiconducting materials, e.g., gallium nitride (GaN) (6.3 ± 0.2 meV) and crystalline silicon (c-Si) (5.4 ± 0.5 meV).^36,37^ We note that the decreasing (increasing) static (dynamic) disorder with an increasing number of cations might indicate an improved structural order (faster ion dynamic) in the triple cation perovskite, although further studies are necessary to make general conclusions in this regard. σ_0_ is found to be close to 2 for all three investigated perovskite systems.

**Figure 3:**
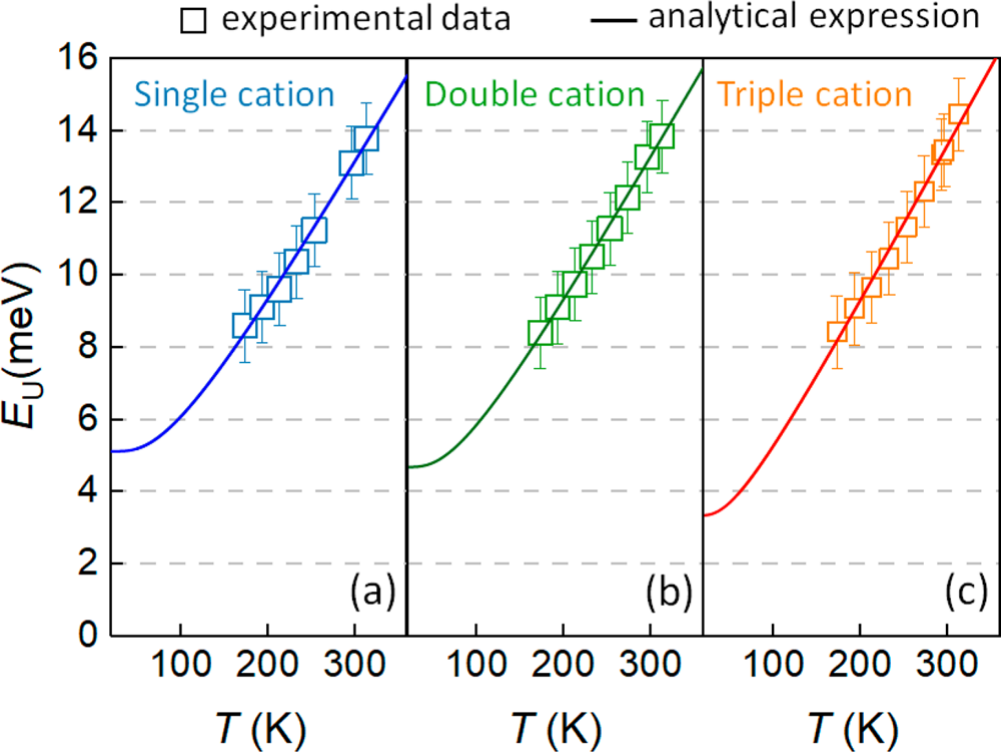
Urbach energy as obtained from *E*_U_^app^ spectra plotted as a function of the temperature for (a) single cation, (b) double cation, and (c) triple cation perovskite solar cells. Symbols are experimental data and solid lines correspond to fits to eq 3. Error bars correspond to the 1 meV uncertainty in Urbach energy determination as a result of optical interference effects. Fit parameters for perovskite systems (a–c) are provided in Table 1.

**Table 1:** Fit Parameters as Obtained for Experimental Data (See Figure 3) According to Eq 3

	*E*_U_(RT) (meV)	*E*_U_(0) (meV)	σ_0_	θ_E_ (K)
single cation	13.0 ± 1.0	5.1 ± 0.5	2.1 ± 0.04	246 ± 29
double cation	13.2 ± 1.0	4.7 ± 0.3	2.03 ± 0.02	220 ± 13
triple cation	13.5 ± 1.0	3.3 ± 0.9	1.94 ± 0.03	150 ± 47

We note that eq 3 is equivalent to an oft-used model based on the Einstein solid model assuming that the disorder in banded semiconductors is caused by lattice phonons.^15,38−40^ The corresponding Einstein temperature is given by θ_E_ = ℏω_v_/*k*. Accordingly, eq 3 can equivalently be expressed in the form of *E*_U_(*T*) = [*E*_0_ + *E*_dyn_(*T*)]/σ_0_, where *E*_0_ = ℏω_v_/2 is the zero-point energy (associated with the lattice phonons) and *E*_dyn_(*T*) = ℏω_v_/[exp(θ_E_/*T*) – 1]. The corresponding equivalent Einstein temperatures are provided in Table 1 for comparison.

It is noteworthy that the Einstein-based model for *E*_U_(*T*) has been further extended empirically to include other effects, such as defect-induced structural disorder,^41^ by adding an additional term *E*_U,struc_ to eq 3. However, these extensions were found to lead to unphysical values in the fittings for the three perovskite systems studied in this work. Instead, a reliable fit can only be obtained for *E*_U,struc_ = 0, suggesting that the static disorder is solely dominated by the quantum mechanical phonon lattice vibrations (zero-point phonon energy), as expected for materials with a nearly perfect crystal structure.^15^

In conclusion, we have demonstrated that optical interference and, thus, film thickness influence the EQE spectral line shapes in the exponential tail region, from which the Urbach energy is determined, leading to an uncertainty in Urbach energy of 1 meV. Taking this uncertainty into account, similar Urbach energies of 13 ± 1 meV at room temperature were obtained on single, double, and triple cation perovskite solar cells from sensitive photocurrent measurements. On the basis of fittings to the temperature dependence of the corresponding Urbach energies, we found static contributions to the Urbach energy in the low-temperature limit are dominated by zero-point phonon energy being as low as 5.1 ± 0.5 meV (single cation), 4.7 ± 0.3 meV (double cation), and 3.3 ± 0.9 meV (triple cation), values that are close to those of c-Si. Our findings suggest nearly perfect structural crystal quality in solution-processed perovskite semiconductors, further broadening their potential application to quantum electronics and devices.

## Supporting Information Available

Details of experimental methods, description of device fabrication, experimental and simulated external quantum efficiency spectra and optical constants, additional information to monochromaticity imperfection, temperature-dependent external quantum efficiency, and corresponding apparent Urbach energy spectra (PDF)
