# Supplementary material for: Static Disorder in Lead Halide Perovskites
Source: J Phys Chem Lett. 2022 Aug 2;13(31):7280–5. doi: 10.1021/acs.jpclett.2c01652 (PMC9376950; doi:10.1021/acs.jpclett.2c01652)
Supplement: Supplementary file 1 — jz2c01652_si_001.pdf [file jz2c01652_si_001.pdf]

Supporting Information

Static Disorder in Lead Halide Perovskites

Stefan Zeiske[1], Oskar J. Sandberg[1], Nasim Zarrabi[1], Christian M. Wolff[2], Meysam Raoufi[3], Francisco Peña-Camargo[3], Emilio Gutierrez-Partida[3], Paul Meredith[1], Martin Stolterfoht[3], and Ardalan Armin[1]**

[1]Sustainable Advanced Materials (Sêr-SAM), Department of Physics, Swansea University, Singleton Park, Swansea SA2 8PP Wales, United Kingdom

[2]EPFL STI IEM PV-LAB, Rue de la Maladière 71b, CH-2002 Neuchâtel 2

[3]Soft Matter Physics Institute of Physics and Astronomy, University Potsdam, Karl-Liebknecht-Str. 24-25, 14476 Potsdam-Golm, Germany

*Email: o.j.sandberg@swansea.ac.uk; ardalan.armin@swansea.ac.uk

Temperature dependent external quantum efficiency (EQE): Sensitive EQE spectra were obtained using a PerkinElmer spectrophotometer (Lambda950) as a light source. The monochromator output light was physically chopped at 273 Hz (Thorlabs MC2000B) and focused on the device under test (DUT) using different optical components. Prior to analyzing the DUT photocurrent response with a Standford Research lock-in amplifier (SR860), the DUT photocurrent was fed into a Femto current pre-amplifier (DCLPCA-200). The EQE system was pre-calibrated using NIST-calibrated silicon and germanium photodiode sensors from Newport (818-UV, 818-IR). A detailed description of the EQE apparatus is provided elsewhere.[1] For temperature dependent EQE measurements, the DUT was mounted in an electrically shielded sample holder (Linkam LTS420 thermal stage) connected to the Linkam T96 temperature controller with integrated LNP96 liquid nitrogen pump.

Current-voltage (*J-V*) characteristics: *J-V* curves were obtained in a 2-wire source-sense configuration with a Keithley 2400. An Oriel class AAA Xenon lamp-based solar simulator was used for illumination providing approximately 100 mW cm^{-2} of AM 1.5G irradiation and the intensity was monitored simultaneously with a Si photodiode. The exact illumination intensity was used for efficiency calculations, and the simulator was calibrated with a KG5 filtered silicon solar cell (certified by the Fraunhofer ISE). The obtained short-circuit current density (J_{SC}) was checked by integrating the product of the corresponding external quantum efficiency (EQE) and the AM 1.5G solar spectrum which matches the obtained J_{SC} within less than 5 %. The temperature of the cell was fixed to 25 °C and a voltage ramp (scan rate) of 67 mV/s was used.

Device fabrication of *p-i-n*-type cells: *Substrates and hole transport layer (HTL):* Pre-patterned 2.5 x 2.5 cm^2 15 Ω/sq. ITO substrates (Automatic Research, Germany) were cleaned with acetone, 3% Hellmanex solution, DI-water and iso-propanol, by sonication for 10 min in each solution. After a microwave plasma treatment (3 min, 200 W), the samples were transferred to a N_2-filled glovebox. For the *p-i-n*-type cells shown in the main text, a PTAA (Sigma-Aldrich) layer with thickness of 8 nm was spin coated from a 1.5 mg mL^{-1} PTAA/toluene solution at 6000 rpm for 30 seconds. After 10 min annealing on a hotplate at 100 °C, the films were cooled down to room temperature and a 60 μL solution of PFN-Br (1-Material, 0.5 mg/mL in methanol) was deposited onto PTAA while the substrate was being spun at 5000 rpm for 20 s resulting in a film with thickness below the detection limit of our AFM (< 5 nm). No further annealing was performed.

Perovskite solutions: The triple cation perovskite solutions were prepared by mixing two 1.2 M $FAPbI_3$ and $MAPbBr_3$ perovskite solutions in DMF:DMSO (4:1 volume ratio, v:v) in a ratio of 83:17 which we call "MAFA" solution. The 1.2 M $FAPbI_3$ solution was prepared by dissolving FAI (722 mg) and PbI_2 (2130 mg) in 2.8 mL DMF and 0.7 mL DMSO which contained a 10 molar% excess of PbI_2. The 1.2 M $MAPbBr_3$ solution was made by dissolving MABr (470 mg) and $PbBr_2$ (1696 mg) in 2.8 mL DMF and 0.7 mL DMSO which contained a 10 molar% excess of $PbBr_2$. Lastly, 40 μL of a 1.5 CsI solution in DMSO (389 mg CsI in 1 mL DMSO) was mixed with 960 μL of the MAFA solution resulting in a nominal perovskite stoichiometry of $Cs_{0.05}(FA_{0.83}MA_{0.17})_{0.95}Pb(I_{0.83}Br_{0.17})_3$. The MAPI solution was prepared by dissolution of MAI powder (Dysol, 0.2) with PbI_2 (TCI America, 1.1 M) in a γ-butyrolactone/dimethyl sulfoxide mixed solvent (7:3 by volume) at 60 °C for 10 min. For the double cation perovskite, 2 different solutions were prepared. The first solution was prepared by dissolving 1.5 M PbI_2 (TCI, 691.55 mg) in 1 mL DMF:DMSO (9:1 volume ratio, v:v). Then a solution containing the cations was prepared by dissolving FAI (Dyenamo, 90 mg), MAI (Lumtec, 6.4 mg) and MACl (Sigma-Aldrich, 9mg) in 1 mL Isopropanol (IPA). Both solutions were left under stirring overnight (14h) to dissolve the materials completely. We note that the addition of MACl improves the crystallization of the perovskite film but evaporates in the final annealing step.

Perovskite film fabrication: The triple cation perovskite was deposited by spin-coating at 5000 rpm for 35 s and 10 s after the start of the spinning process, the spinning substrate was washed with 300 μL ethylacetate for approximately 1 s (the anti-solvent was placed in the center of the film). We note, that by the end of the spinning process the perovskite film turned dark brown. The perovskite film was then annealed at 100 °C for 1 h on a preheated hotplate where the film turned slightly darker. The MAPI solution (80 μL) was spin-coated at 1000 rpm for 5 s followed by 3000 rpm for 80 s. 100 μL toluene was added dropwise after 40 s to form a transparent perovskite film. After the spin coating, the films were dried for 2 min in the glovebox at room temperature until the films changed their color from yellow to light brown. The MAPI perovskite layers were then subsequently annealed at 100 °C on a hotplate where the films turned black immediately. For the double cation perovskite, a 2-step interdiffusion method was employed. The first step was the deposition of the PbI_2 layer. This was achieved by dropping 150 μL of the lead iodide solution in a N_2-filled glovebox and spin-coating at 1500 rpm for 30 s, the films were then annealed at 70 °C for 1 min resulting in a yellow-transparent layer. We note that if the PbI_2 layers are left for too long before proceeding to the next step, the lead

iodides appearance would change to a rougher yellow color and the performance of the complete devices would be substantially lower. Afterwards the formation of the perovskite was achieved by dropping 150 μL of the cation solution on top of the lead iodide layer which turned the film into a brown-transparent layer, then rapidly starting the spin-coating at 2500 rpm for 30 s, which resulted in a red film. This was followed by an annealing step at 150 °C for 15 min outside of the glovebox under ambient conditions (at a relative humidity of 25%) which resulted in a dark brown or black perovskite film. For the second-annealing step, a transport-box was used to transfer the samples out of the glovebox and in order to keep the layers in a nitrogen atmosphere immediately before the annealing on a preheated plate (150 °C). We note that as soon as the perovskite layers get into contact with air, the crystallization and a phase-change process starts, and we noticed that a rapid transfer to the hotplate directly after opening the transport-box was beneficial for the cell performance. A Supplementary Video is available on the publishers webpage of ref. [2] that shows the critical fabrication steps.

Electron transport layer (ETL) and Top Contact: After annealing, the samples were transferred to an evaporation chamber where fullerene-C_{60} (30 nm), 2,9-Dimethyl-4,7-diphenyl-1,10-phenanthroline BCP (8 nm) and copper (100 nm) were deposited under vacuum ($p = 10^{-7}$ mbar). The overlap of the copper and the ITO electrodes defined the active area of the pixel (6 mm^2).

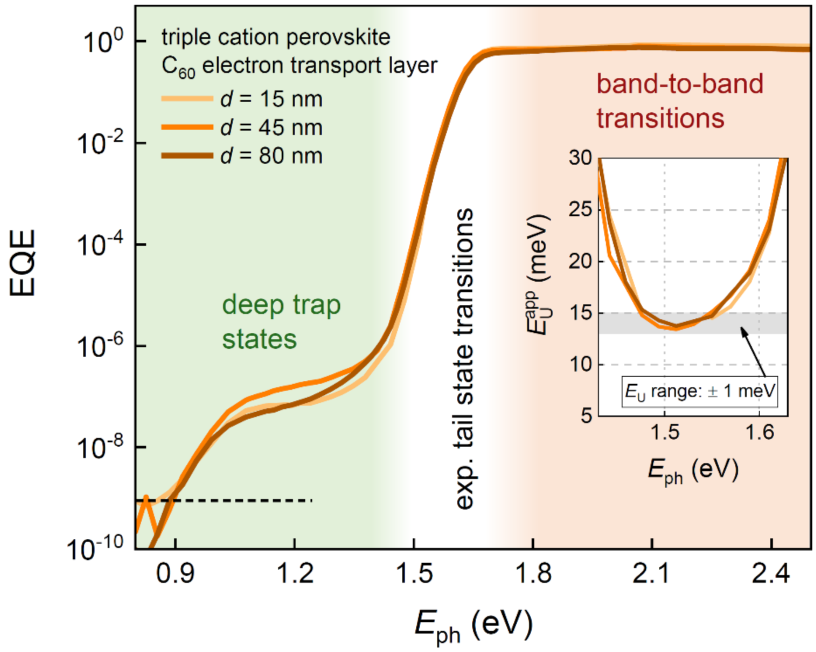

Figure S1 Experimentally obtained external quantum efficiency (EQE) spectra of 360 nm thick triple cation perovskite solar cells having different C_{60} electron transport layer thicknesses. The vertical dashed line indicates the EQE noise floor. The inset shows the apparent Urbach energy spectra (E_U^{app}) in the exponential region calculated using Equation (2) provided in the main text. A thickness dependence is seen leading to an uncertainty in Urbach energy (E_U) determination of ± 1 meV. Note that these devices were fabricated from the same precursor with the same recipe and in the same fabrication round as devices shown in Figure 1b in the main text.

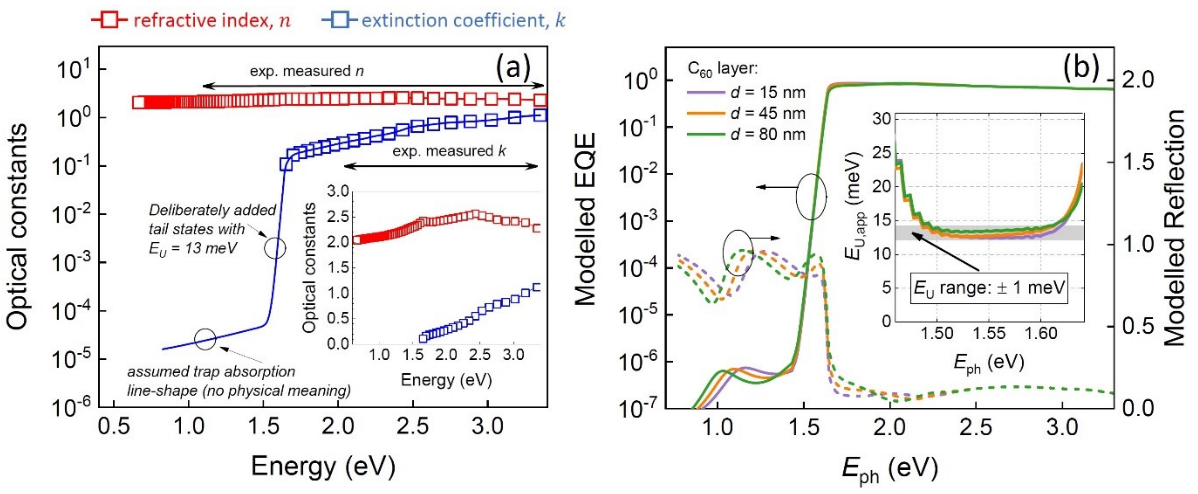

Figure S2 (a) The optical constants of the perovskite layer assumed for the simulations. The refractive index (n) is based on the experimentally measured spectrum of triple cation perovskite (red symbols). For the extinction coefficient (k), experimentally measured values are used for energies above the bandgap (blue symbols); for energies below the bandgap an exponential absorption corresponding to a known Urbach energy of 13 meV is assumed (solid blue line), while at energies below 1.5 eV a "trap induced" absorption line shape is assumed (solid blue line). The inset shows the experimentally obtained optical components expressed in a linear scale. **(b)** Modelled EQE (left axis) and Reflection spectrum (right axis) using the optical constants shown in (a) as input parameters. The inset shows the apparent Urbach energy spectra calculated along Equation (2) provided in the main text.

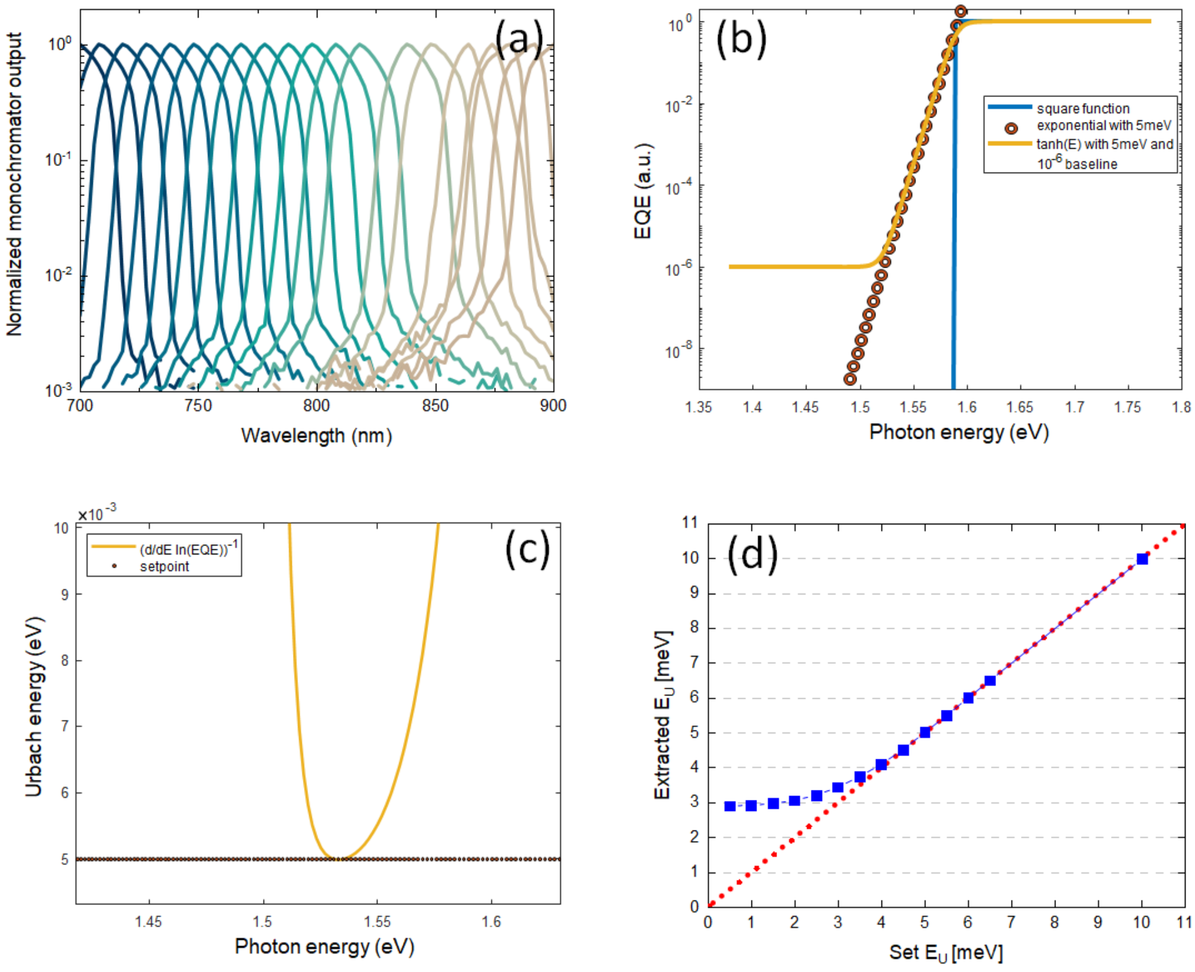

Figure S3 (a) Normalized output of the external quantum efficiency (EQE) monochromator system measured at different set-wavelengths between 700 – 900 nm: a finite linewidth (i.e., full width to half maximum, or FWHM) of ~ 8 nm is revealed. The monochromaticity imperfection means that even a square function shape bandgap will appear to have an artificially broadened onset with a finite Urbach energy, E_{U}. **(b)** Different absorption onsets plotted as a function of photon energy: square function (solid blue line), exponential with $E_{\mathrm{U}} = 5$ meV (red circle symbols), and $A = 0.5 \times \left[\tanh\left(\frac{E-E_{\mathrm{g}}}{2E_{\mathrm{U}}}\right) + 1\right]$ (solid yellow line), where A is the absorption, E the photon energy, and E_{g} denotes the bandgap energy. **(c)** Apparent Urbach energy spectrum (sold yellow line), as estimated for the measured monochromator output (i.e., FWHM ~ 8 nm) and a 5 meV *set* absorption onset, revealing a minimum resolvable E_{U} of exactly 5 meV (vertical dotted line). **(d)** Minimum resolvable Urbach energy (blue symbols), as obtained from the apparent Urbach energy spectra, plotted as a function of *set* absorption onsets: a saturation of extracted Urbach energies to ~ 3 meV is observed defining the lower limit of experimentally observable E_{U} values. The red symbols correspond to the ideal case, where no monochromaticity imperfection (corresponding to a FWHM of 0 nm) is present. A further reduction of the lower limit of observable E_{U} can be achieved by reducing the monochromaticity imperfection (i.e., the linewidth of the monochromator output).

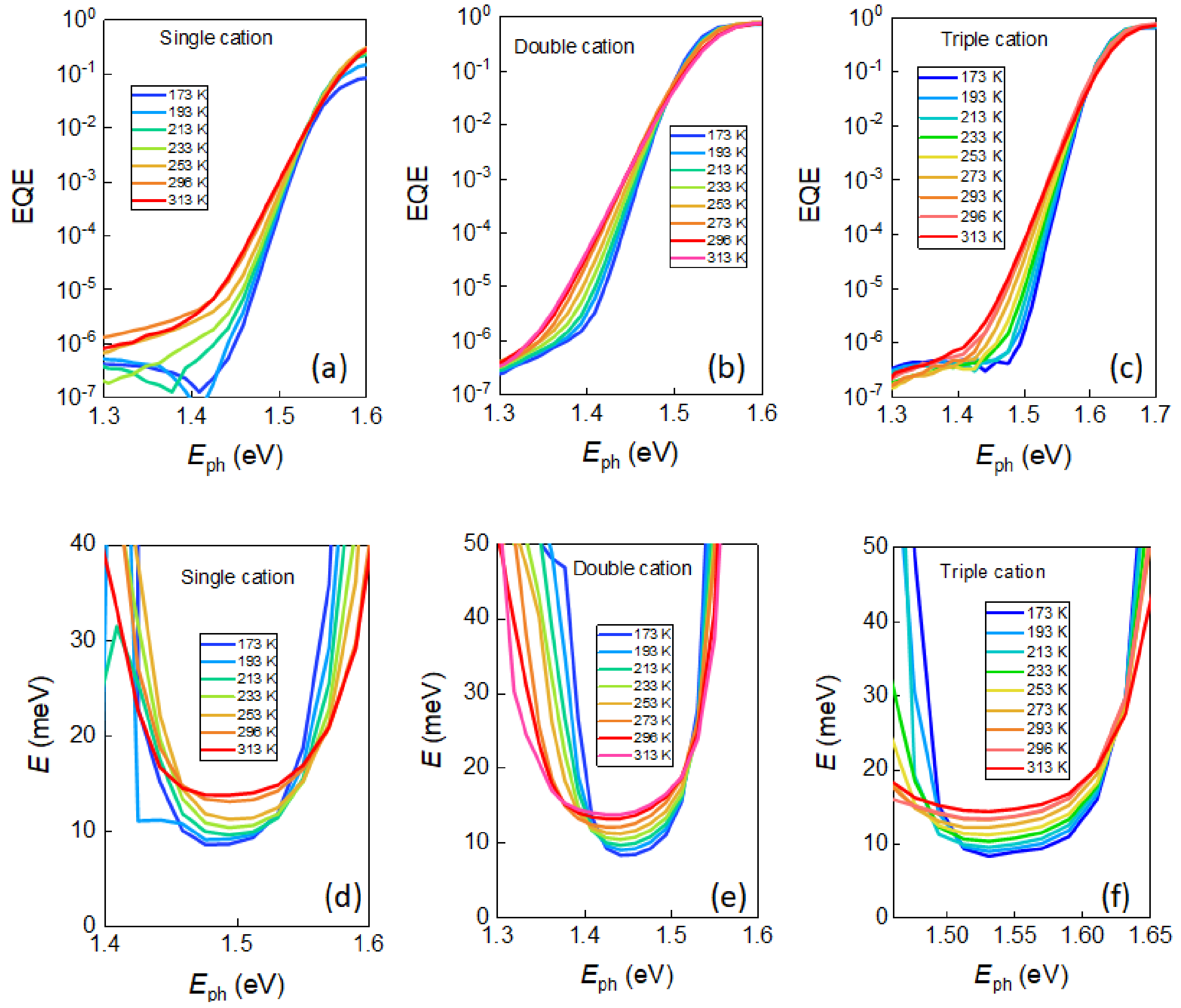

Figure S4 External quantum efficiency (EQE) spectrum of **(a-c)** single, double, and triple cation perovskite solar cell plotted as a function of photon energy and compared for different temperatures. **(d-f)** Repetition of panel (a-c), but apparent Urbach energy spectra plotted as a function of photon energy and compared for different temperatures. The Urbach energy spectra were calculated according to Equation (2) shown in the main text.

Supplementary References:

(1) Zeiske, S.; Kaiser, C.; Meredith, P.; Armin, A. Sensitivity of Sub-Bandgap External Quantum Efficiency Measurements of Solar Cells under Electrical and Light Bias. *ACS Photonics* **2019**, *7*, 256–264.

(2) Gutierrez-Partida, E.; Hempel, H.; Caicedo-Dávila, S.; Raoufi, M.; Peña-Camargo, F.; Grischek, M.; Gunder, R.; Diekmann, J.; Caprioglio, P.; Brinkmann, K. O.; Köbler, H.; Albrecht, S.; Riedl, T.; Abate, A.; Abou-Ras, D.; Unold, T.; Neher, D.; Stolterfoht, M. Large-Grain Double Cation Perovskites with 18 Ms Lifetime and High Luminescence Yield for Efficient Inverted Perovskite Solar Cells. *ACS Energy Lett.* **2021**, *6*, 1045–1054.
